# Isolation of Three Lycorine Type Alkaloids from *Rhodolirium speciosum* (Herb.) Ravenna Using pH-Zone-Refinement Centrifugal Partition Chromatography and Their Acetylcholinesterase Inhibitory Activities

**DOI:** 10.3390/metabo10080309

**Published:** 2020-07-28

**Authors:** Diana Isabel Correa, Edgar Pastene-Navarrete, Luis Bustamante, Marcelo Baeza, Julio Alarcón-Enos

**Affiliations:** 1 Laboratorio de Farmacognosia, Dpto. de Farmacia, Facultad de Farmacia, Unidad de Desarrollo Tecnológico, UDT. P.O. Box 237, Universidad de Concepción, PC4030000 Concepción, Chile; dianacorrea0114@gmail.com; 2 Laboratorio de Síntesis y Biotransformación de Productos Naturales, Dpto. Ciencias Básicas, Universidad del Bio-Bio, PC3780000 Chillan, Chile; 3 Dpto. de Análisis Instrumental, Facultad de Farmacia, Universidad de Concepción, PC4030000 Concepción, Chile; lbustamante@udec.cl; 4 Dpto. Botánica, Facultad de Ciencias Naturales y Oceanográficas, Universidad de Concepción, PC4030000 Concepción, Chile; cbaeza@udec.cl

**Keywords:** acetylcholinesterase, amaryllidaceae alkaloids, gas chromatography, pH zone refining centrifugal partition chromatography, mass spectrometry, nuclear magnetic resonance

## Abstract

Preparative separation of three lycorine type alkaloids from *Rhodolirum speciosum* (Amaryllidaceae) was successfully carried out using pH-zone-refinement centrifugal partition chromatography (CPC) using the solvent system methyl-tert-butyl ether/acetonitrile/water (4:1:5, *v*/*v*/*v*) in descending mode. Using this system, Alkaloid 1 (165.7 mg, 88.2%, purity), 2 (60.1 mg, 97.7% purity) and 3 (12.3 mg, 84.4% purity) were obtained in one step. For structure elucidation, the pure alkaloids were subjected to spectroscopy analysis using nuclear magnetic resonance experiments (^1^H-NMR, ^13^C-NMR) and gas chromatography coupled with mass spectrometry (GC-MS). Alkaloids 1, 2, and 3 were identified as 1-*O*-acetyl-5,6-dehydrolycorine, 1-*O*-acetyl-lycorine, and 1,2-*O*-diacetyl-5,6-dehydrolycorine, respectively. The acetylcholinesterase inhibitory activity of these alkaloids was IC_50_ 151.1 μg/mL, IC_50_ 203.5 μg/mL, IC_50_ 470.0 μg/mL, and IC_50_ 17.1 μg/mL, respectively.

## 1. Introduction

**Figure 1:**
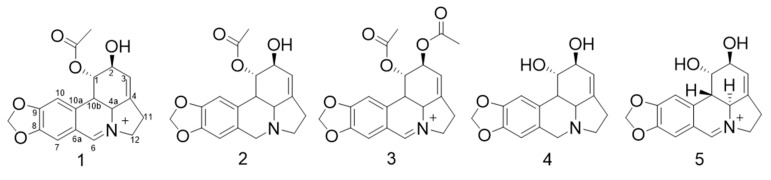
Chemical structure of lycorine-type alkaloids (**1**, **2**, and **3**) isolated from *Rhodolirium speciosum*, lycorine (**4**), and 5,6-dehydrolycorine (**5**).

Amaryllidaceae are perennial bulbous herbaceous plants distributed worldwide on coasts, in forests and highlands, as well as in tropical and subtropical areas. In Chile they are represented by 11 genera and 35 species, among which is the endemic species *Rhodolirium speciosum* (Herb.) Ravenna [1]. These plants are found within the twenty families of the most important alkaloid producing species. Isoquinoline type alkaloids are exclusive to this family and are biogenetically related through specific oxidative phenolic couplings of the biogenetic precursor *O*-methylnorbelladine derived from the amino acids l-phenylalanine and l-tyrosine. In the biosynthetic pathway of these alkaloids more than six intermediates are formed, some resulting from resonant structures. Furthermore, alkaloids such as homolicorine, galanthine, norpluvin, noroxopluvine, and caranine, are obtained as biosynthetic products of the same route leading to lycorine and homolycorine (Bastida et al. 2011) [2]. Although the first four enzymatic stages responsible for the synthesis of many lycorin derivatives are known in some depth, the last step of the biosynthesis is poorly characterized. For instance, the synthetic pathway to lycorine-type alkaloids requires the formation of a methyledioxy bridge in norpluviine to form caranine, and then lycorine can be obtained via hydroxylation. So far, the genes encoding the enzymes responsible for the modifications that may occur on these final structures are unknown. (Desgagné-Penix, 2020) [3]. Previous studies in different species of the *Rhodolirium* genus report between 20 and 30 alkaloids, among which are galanthamine, lycorine, heamanthamine, and undulatine, together with a high number of unidentified alkaloids [4,5]. There is great evidence of the pharmacological potential of these metabolites, such as anticancer [6], cytotoxicity [7], antibacterial [8], and neuroprotection activity [5]. The potential for inhibition of acetylcholinesterase (AChE) that Amaryllidaceae alkaloids have is well known; enzyme inhibition suppresses hydrolysis of acetylcholine, a neurotransmitter that mediates the synaptic activity of the nervous system. Galanthamine is one of the FDA approved medications, used for the treatment of cognitive decline treatment of Alzheimer’s disease (AD) in mild to moderate stages, so alkaloids with similar carbon nucleus could be molecules with promising AChE inhibition activity and neuroprotection [9]. Approximately 500 alkaloids have been isolated from this family of plants [10]. However, due to the low content, structural diversity, and pH-dependent solubility, their isolation and purification requires large amounts of plant material and therefore higher solvent consumption. Solid support- based chromatography is the most used to isolate alkaloids, however, irreversible adsorption and stationary phase limitations decrease the efficiency of the purification process [11]. Displacement centrifugal partition chromatography (CPC) strategy has been increasingly used to isolate and purify large amounts of bioactive compounds [12]. This protocol is restricted to ionizable compounds, which have a dramatic difference in solubility between their ionized and neutral forms. In this context, pH-zone-refinement CPC is the preferred methodology for the isolation of alkaloids. Previous studies have reported the efficiency of this technique for isolating isoquinoline alkaloids such as isocorydine, corydine [13], huperzines A y B [14], bicuculline, protopine [15], magnoflorine, berberine [16], and palmatine [17]. Therefore, Amaryllidaceae alkaloids are good candidates to be separated and purified by pH-zone-refinement CPC. This support-free liquid–liquid partition and displacement chromatography has many advantages such as; high sample loading capacity with minimal irreversible adsorption or stationary phase saturation and high selectivity. These advantages allow the separation of compounds with very similar structures in high purity and low solvent consumption compared to conventional preparative techniques [18]. So far, this is the first report on the use of pH-zone-refinement CPC for the isolation and purification of isoquinoline alkaloids from *R. speciosum*. Thus, herein we report the development of a pH-zone-refinement CPC method for preparative isolation and purification of three major alkaloids from *R. speciosum* (Figure 1). Their identification was performed by means of chromatographic and spectroscopic methods. Also, we evaluate the in vitro AChE-inhibiting properties of these isolated compounds.

## 2. Results and Discussion

### 2.1. GC-MS Analysis of Rhodolirium Speciosum

Plants of the Amaryllidaceae family are an important source of alkaloids, which have varied biological activities. The literature reports countless works which, through the use of LC-ESI-MS-MS or GC-MS-MS, give an account of the existence of these alkaloids in the different genera of this family, reaching the presence of 30 to 40 alkaloids in a plant. In a previous work with *Rhodophiala pratensis* through GC-MS our group reported 34 alkaloids, achieving the identification of 24 of them [5]. In the present work, the preliminary analysis by GC-MS of crude alkaloid extract of *R. speciosum* revealed the presence of alkaloids with lycorine, 5,6-dehydrolycorine, galanthamine, haemanthamine, and montanine skeleton, and a set of not identified alkaloids, although their fragmentation pattern suggests that they are lycorine derivatives (Figure 1, Table 1).

### 2.2. Isolation of Alkaloids by Means of pH-Zone-Refinement CPC

The pH-zone-refinement CPC technique is based on the displacement of molecules with difference solubility between the ionized and neutral form. According to Ito [19] an optimal biphasic system for pH-zone-refining CPC must give K_basic_ >>1 and K_acid_ << 1 in descending mode. An important advantage of this separation methodology is that high loads of extract can be introduced in the CPC system giving irregular rectangular peaks with impurities confined in the initial and outside boundaries of the main peak. This feature allows large-scale separations of ionizable compounds depending on the pH variation. So, in this methodology the selectivity of separation (Ka′) is largely controlled by Ka × KD, where Ka is the acidity constant and KD is the distribution constant [14]. Amaryllidaceae alkaloids containing a secondary, tertiary or even quaternary nitrogen atom, are moderately weak bases with variable pKa between 4.0 and 10.0, so that their basic neutral forms and acid protonates are solubilized in organic and aqueous phases, respectively. Due to the advantages of pH-zone-refinement CPC, the large-scale purification of compounds with low yields is possible. So, these advantages encouraged us to select pH-zone-refinement CPC as a preparative separation strategy for this class of alkaloids. As mentioned above, for the present application to alkaloids, a suitable two-phase solvent system was necessary which should provide ideal distribution constants (KD) values in both acidic and basic conditions as well as good solubility of the samples in the solvent system. We started the evaluation using different combination of the previously reported biphasic solvent system *n*-Hept/EtOAc/*n*-PrOH/W (10:30:15:45 *v*/*v*/*v*/*v*) with 15 mM TEA and variable concentrations of formic acid, acetic acid, and HCl (Table 2). Under such conditions it was observed that alkaloids cannot be separated properly. For instance, when 6 mM HCl was used, alkaloids were retained in the stationary phase (Figure S1) with retention times too long (>210 min).

In the case of solvent system *n*-Hept/EtOAc/*n*-PrOH/W (10:30:15:45 *v*/*v*/*v*/*v*) with 6 mM acetic acid, and 3 mM formic acid, KD values only could be estimated for compound 1 and 2 and they were not good enough for our purpose. This problem caused co-elution of alkaloids and neutral compounds in the pH-zone-refinement CPC experiment (Table 3, Figures S2 and S3). Therefore, we evaluated a binary two-phase solvent system composed of MtBE–W (1:1, *v*/*v*) which has been used for different types of compounds [20]. However, with this solvent system the alkaloids showed solubility problems. Afterwards, we prepared the biphasic solvent system MtBE/ACN/W (4:1:5, *v*/*v*), which had been used previously for alkaloid separation [21,22]. Interestingly, the solubility of the sample was substantially improved by adding ACN to the above mentioned solvent system. So, when this solvent was used with 12 mM TEA with 6 mM formic acid, the separation was improved and alkaloid 1 could be purified (t_R_ = 15–20 min). However, alkaloids 2 and 3 co-eluted as a single rectangular shape peak around 30–40 min (Figure S4).

Nevertheless, this system can be optimized in order to obtain Kacid and Kbase values sufficiently different to guarantee an adequate separation of the targeted alkaloids. Thus, as shown in Table 4, among the different conditions tested, the solvent system MtBE/ACN/W in the proportions 4:1:5, *v*/*v*/*v* with 6 mM formic acid and 15 mM TEA gave Kacid 33.76 and Kbase 1.35 values for alkaloid 1 and Kacid 48.65 and Kbase 0.74 values for alkaloid 2, while for alkaloid 3 Kacid 0.43 and Kbase 12.52 were calculated. This system seems more suitable for purify main alkaloids, albeit higher acid concentrations could be used to improve the purification of other minor alkaloids. However, in order to make the separation in one-step, we decided on the MtBE/ACN/W with 6 mM formic acid and 15 mM TEA system.

**Figure 2:**
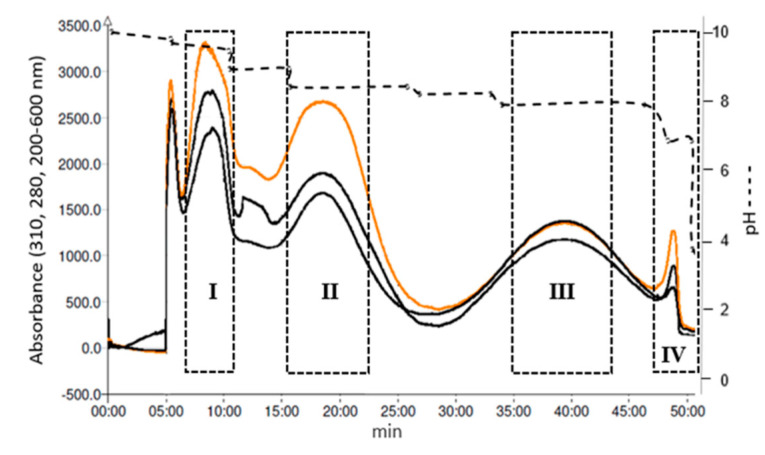
Illustrative pH-zone-refinement Centrifugal Chromatography separation of main alkaloids from *Rhodolirium speciosum*. Conditions: stationary phase, the upper phase of MtBE/ACN/W (4:1:5, *v*/*v*/*v*); detection, 280 nm (orange line); flow-rate, 12 mL/min; revolution speed, 1800 rpm, 6 mM formic acid in lower stationary phase and 15 mM TEA in upper phase, sample loading 556.2 mg. Retention of stationary phase was 76%.

**Figure 3:**
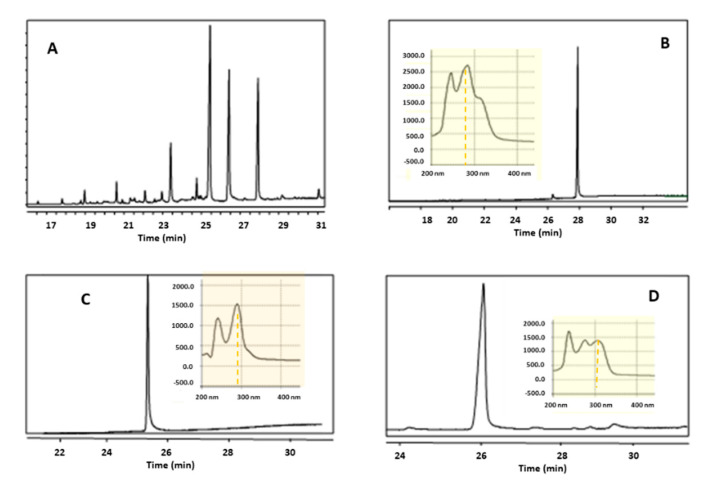
Representative GC-MS analysis of (**A**) crude alkaloids of *Rhodolirium speciosum* and CPC isolated main compounds. (**B**) Alkaloid 1, (**C**) Alkaloid 2 and (**D**) Alkaloid 3. UV-VIS spectra depicted in the insets were obtained directly from the preparative PDA detector of the CPC apparatus.

Figure 2 shows the preparative pH-zone-refinement CPC separation of 556.2 mg crude of alkaloids from *R. speciosum* sample using the selected solvent system. The retention of stationary phase was 76%. In Figure 2, Zone I has a mixture of minor alkaloids and impurities poorly resolved. As the pH decreases three clearly delimited zones can be observed named F II, III, and IV. After preliminary HPLC analysis of each tube, those with identical composition were gathered, concentrated, and cleaned to eliminate TEA. Afterwards, CPC fractions with alkaloids were re- analyzed by HPLC and GC-MS in order to check the purity of each compound. Figure 3 shows the chromatograms of crude alkaloid extract, isolated alkaloids 1–3 and their respective UV spectra (taken from a CPC PDA detector). After GC-MS analysis, single peaks (alkaloids 1, 2, and 3) with minor neutral impurities were observed in F II-IV. As a result, 65.7 mg alkaloid 1 (88.2% purity), 50.1 mg alkaloid 2 (97.7% purity), and 12.3 mg alkaloid 3 (84.4% purity), were obtained in a single step separation from zone II-IV, respectively (Table 4). The elucidation of the chemical structures for each alkaloid isolated from *R. speciosum* using pH-zone-refinement CPC was carried out by positive EI-MS, ^1^H NMR and ^13^C NMR (see Section 2.3).

The isolation of alkaloids by pH zone refinement is not yet so widespread among natural products researchers, although several examples of its potential can be mentioned. In a work of Zhu et al., it was reported that it enabled the purification of 53.7 mg of stepharanine, 28.1 mg of columbamine, 150.6 mg of jatrorrhizine,169.8 mg of palmatine, and 157.2 mg of berberine starting from 3.0 g of alkaloid extract of *Caulis mahoniae* [23]. That means that around 19% of the crude extract corresponds to these five alkaloids. In our study, we loaded 556.2 mg of the crude alkaloid extract of *R. speciosum* in the CPC device obtaining 65.7 mg of alkaloid 1, 50.1 mg of alkaloid 2 and 12.3 mg of alkaloid 3. This means that 23% of the crude alkaloid extract of *R. speciosum* corresponds to these three alkaloids. Importantly, it must be considered that this productivity is approximately per hour of run. On the other hand, Sun et al. [24], reported that the sample loading capacity of pH- zone- refinement Counter Chromatography was 150-fold greater than other chromatographic methodologies used to isolate *Lycoris radiata* alkaloids. In a work of Huang and coworkers [25], 180 kg of *L. radiata* were necessary to produce 1.4 kg of alkaloid extract in order to isolate mg to grams of six alkaloids with productivities ranging from 0.00057 to 1.92% using conventional methods of isolation. In another study, Kotland et al. [26] demonstrated that pH-zone-refinement CPC for the alkaloids isolated from *Catharanthus roseus* could be scaled-up using CPC equipment with rotor capacity from 25 mL to 2 L. Therefore, in an FCPC25 (25 mL rotor capacity), 3.38 g of extract were loaded whereas in an FCPE300 (300 mL rotor capacity), 27 g were injected using an 80 mL sample loop. Finally, these authors used an FCPC D2 L (2 L rotor capacity) to separate 150 g of crude extract achieving the outstanding processing capacity of 4 kg crude extract per day and a productivity of 170 mmol of alkaloids (sum of four main *C. roseus* alkaloids)/L/h. To isolate Amaryllidaceae alkaloids, extractive processes often have to be carried out starting from a large amount of sample. An additional problem and added to the fact that the concentrations of these alkaloids are very low in the bulbs, is that their collection is laborious and not very ecological. For example, several kg of sample must be collected to compensate for the losses associated with the different liquid–liquid extraction processes and many steps of separations on solid chromatographic supports. In another work of Gonring-Salarine et al. [27], 6.7 kg of *Worsleya procera* bulbs were necessary to obtain 7.0 g of alkaloid extract to isolate around 505 mg of lycorine (7.21%), after several purification steps. It is important to point out that the strategy developed by us using pH-zone-refinement CPC not only allows the introduction of high loads of sample but also saves time. Since purification could be done in one or two steps maximum, sample losses by irreversible retention were minimum, thereby larger amounts of alkaloids could be obtained with high purity levels.

### 2.3. Isolated Alkaloids

**Figure 4:**
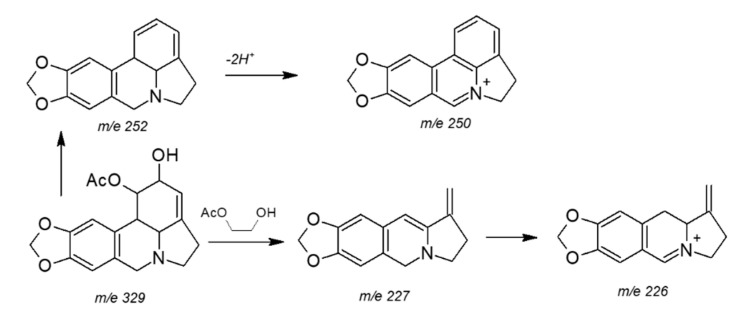
Proposed MS fragmentation pattern of the alkaloids isolated from *Rhodolirium speciosum*.

Alkaloids 1, 2, and 3 were identified by comparison of their EI-MS, NMR and chromatographic data with authentic compounds isolated from other Amaryllidaceae species and comparison with the literature [28,29,30]. Alkaloid 1 was obtained as a yellow amorphous powder. The EI-MS afforded a quasimolecular ion peak at *m*/*z* 327 (calcd. for C_18_H_18_NO_5_^+^, 328.33867), corresponding to the molecular formula C_18_H_18_NO_5_^+^, and shows a fragmentation patterns typical of lycorine-type alkaloids (Figure 4). Its UV absorption at λmax 374, 309, 253, and 212 nm showed an extended chromophore and a methylenedioxyl substituted benzene ring. The IR absorption bands at 3.410, 3.355, 1.645, 1.605, and 923 cm^−1^ indicated OH groups and phenyl functions. The ^1^H-NMR spectrum of alkaloid 1 (Table 5), exhibited two singlets for two para-located aromatic protons at δH 7.07 (H-8) and 7.51 (H-11), a methylenedioxy signal at δH 6.11 and a downfield singlet corresponding to the proton of an iminium salt (δH 8.47) [19]. The ^13^C-NMR spectrum showed 18 carbon signals [OCH_2_O × 1, CH_2_ (sp^3^) × 2, CH (sp^3^) × 4, CH (sp^2^) × 4 and C (sp^2^) × 6, and CH_3_ (sp^3^) x 1, (Table 5). The above data suggested that alkaloid 1 is an Amaryllidaceae alkaloid similar to lycorine [24,25], except for an imine moiety located between *n*-5 and C-6 (δC 168.15) in compound 1, as supported by HMBCs of δH 8.47 (H-6) with δC 60.62 (C-4a), 109.64 (C-7), 136.93 (C-10a) and 55.77 (C-12). The relative configuration of H-4a and H-10b in the Amaryllidaceae alkaloids isolated from the genus Lycoris were always α- and β-orientations, respectively [31,32]. The relative configuration of alkaloid 1 was elucidated by a ROESY experiment. The ROESY correlations of H-10b/H-1 and H-4a/H-2 indicated the β-orientation of H-1 and α-orientation of H-2 and are consistent with those reported by Kotera et al. [32]. Therefore, alkaloid 1 was identified as 1-O-acetyl-5,6-dehydrolycorine.

Alkaloid 2 was obtained as a yellow amorphous powder. The EI-MS afforded a quasimolecular ion peak at *m*/*z* 330 (calcd. for C_18_H_19_NO_5_^+^, 329.347000). The ^1^H-NMR spectrum of alkaloid 2 (Table 5), differed with regard to alkaloid 1 in the absence to downfield singlet corresponding to the proton of an iminium salt. Analysis of the ^13^C-NMR and DEPT spectra revealed the presence of one methyl carbon, four methylene carbons, and seven methine carbons. Therefore, alkaloid 2 was identified as 1-O-acetyl-lycorine. Alkaloid 3 was obtained as a yellow amorphous powder. The EI-MS afforded a quasimolecular ion peak at *m*/*z* 370 (calcd. for C_19_H_18_NO_5_^+^, 370.375351)

In the same way, compound 3 has signals whose displacement is related to alkaloid 1, the difference is produced by the presence of one additional acetate group. The ^1^H-NMR spectrum of alkaloid 3 (Table 5), exhibited two singlets for two para-located aromatic proton at δH 7.49 (H-7) and δH 6.62 (H-10), a methylenedioxy signal at δH 6.62, two singles at 2.67 and 2.11 ppm, which integrate as three protons corresponding to two methyl groups, and a downfield singlet corresponding to the proton of an iminium salt (δH 8.55). Analysis of the ^13^C-NMR and DEPT spectra revealed the presence of two methyl carbons, three methylene carbons, and eight methine carbons. Further analysis of the two and three bond cross-correlations revealed that H-2, H-4a, H-10b, H-11, and H-12 all coupled with C-4. Therefore, alkaloid 3 was identified as 1,2-O-diacetyl-5,6-dehydrolycorine. In the case of 1-acetyl-5,6-dehydrolycorinium (alkaloid 1) and 1,2-diacetyl-5,6-dehydrolycorinium (alkaloid 3), only the base structure of 5,6-dehydrolycorine was reported by Bin et al. [33]. Until now, these acetyl-derivatives of dehydrolycorine have not been isolated.

### 2.4. Acethylcholinesterase Inhibition Assay

**Figure 5:**
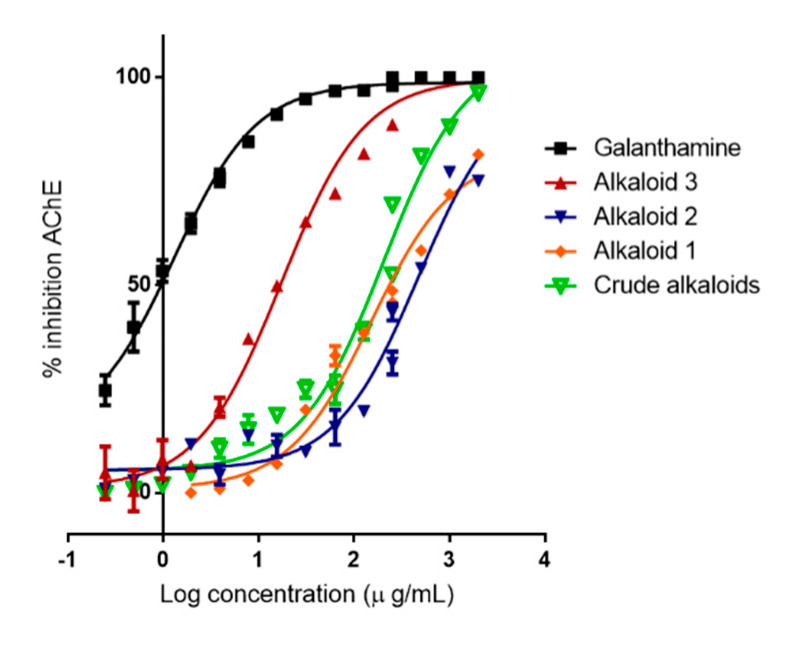
AChE inhibitory activity curves of isolated lycorin derivatives, crude alkaloids and the reference compound galanthamine.

The acetylcholinesterase inhibition curves for *R. speciosum* alkaloid crude and the three isolated lycorine alkaloids are shown in Figure 5, alkaloid 3 and crude alkaloid extract have the same efficacy as the clinical inhibitor galanthamine (IC_50_ 1.3 μg/mL), however this is more potent than alkaloid 3 (IC_50_ 17.14 μg/mL) and crude alkaloid extract (IC_50_ 151.1 μg/mL), respectively. Alkaloid 1 (IC_50_ 203.5 μg/mL) and alkaloid 2 (IC_50_ 470.0 μg/mL), presents inhibitory activity on of AChE, but their potency and efficacy are lower than alkaloid 3 and crude alkaloid extract. Alkaloid 1 with two acetylated substituent groups evidently showed greater activity than the other two alkaloids and that the crude alkaloid extract presented a moderate to good inhibition, this is consistent with that reported by McNulty et al. [34] and other authors [35,36].

## 3. Materials and Methods

### 3.1. Reagents and Solvents

Methyl-tert-butyl ether (MtBE), *n*-heptane (Hep); ethyl acetate (EtOAc); *n*-propanol (*n*-PrOH); acetonitrile (ACN), hydrochloric acid (HCl), triethylamine (TEA) used for CPC were analytical grade and purchased from Merck (Darmstadt, Germany). Methanol, water, and ACN used for HPLC analyses was of chromatographic grade (Darmstadt, Germany).

### 3.2. Plant Material

*Rhodolirium speciosum* bulbs were collected in the flowering period (October–November) in Coronel location (Santa Juana), in the Bio-Bio region, at coordinates 37°10′S 72°58′ W, with collecting voucher C. Baeza 4350 (CONC). The bulbs were cleaned with distilled water and neutral soap, chopped, lyophilized and stored at −80 °C.

### 3.3. Extraction and Isolation

Lyophilized bulbs (75.0 g) were ground in a blender to a fine powder. The powdered plant material was macerated for 24 h with methanol. Then, methanol extract was sonicated in an ultrasonic cleaner (42 kHz, 70 W; Branson ultrasonic corporation, Brookfield, CT, USA) for 30 min, and filtered. The filtrate was concentrated under vacuum to dryness to obtain the methanol extract. The powdered methanol extract (13.6 g) was dissolved in 50 mL of 2% H_2_SO_4_ and successive extractions were carried out with ethyl acetate (4 × 40 mL). The lower aqueous layer was adjusted to pH 10 with 25% NH_4_OH and extracted successively with chloroform (4 × 40 mL). The remaining ethyl acetate fractions were dried and the residue was extracted again with 10 mL of 2% H_2_SO_4_ repeating the procedure described above. The combined extracts were dried under vacuum to afford crude alkaloids and stored in an air tight container in a desiccator.

### 3.4. CPC Apparatus and Separation Procedures

The separation was performed in a Spot-CPC-250-B Bio-Extractor 250-L centrifugal partition chromatograph (Gilson, France) with a total column capacity of 250 mL. The solvents were pumped by a SPOT-PREP II system (Armen, France), equipped with a quaternary pump, PDA detector and fraction collector. The biphasic solvent systems tested in the present work for the alkaloid separations were mixtures of methyl tert-butyl ether (MtBE)/acetonitrile (ACN)/water (W) and *n*-heptane (Hep)/ethyl acetate (EtOAc)/*n*-propanol (*n*-PrOH)/water (W) [15]. Solvent systems were vigorously shaken and once two phases were separated, triethylamine (TEA) was added as a retainer to the organic phase (upper) and acid (formic, acetic or HCl) was added as a displacer to the aqueous phase (lower) at different concentrations (Table S1). The organic upper phase was used as the stationary phase and the aqueous lower phase was used as the mobile phase. To carry out the refinement by pH-zone-refinement CPC, the upper organic phase was first pumped at a flow rate of 30 mL/min and the column rotated at 500 rpm without phase equilibrium. Crude *Rhodolirium speciosum* alkaloid extract (556.2 mg) was dissolved in 5 mL of the upper phase without retainer and 5 mL of the lower phase with displacer and loaded through a 10 mL sample loop. The lower phase was pumped into the system in descending mode at a flow rate of 12 mL/min increasing the rotation speed up to 1800 rpm. Fractions (25 mL, 26 tubes) were collected, and monitored with a scan of 200–600 nm and wavelengths 280 and 310 nm. Additionally, the changes in the pH of the effluent were monitored during each run.

### 3.5. HPLC Analysis of CPC Fractions

The collected fractions were concentrated under vacuum to dryness and 25% NH_4_OH was added until pH 10, then extracted with chloroform (3 × 5 mL), and concentrated under vacuum to dryness. All fractions were reconstituted in 10 mL of mobile phase and were analyzed by liquid chromatography on a Waters liquid chromatograph (Waters Corporation, Milford, MA, USA), Alliance 2695 model; the system consisted of a quaternary pump LC20AT, column oven CTO-20AC, degassing unit DGU-20AR 5R, detector UV-VIS Waters dual-λ model 2487 (USA), and an autosampler SIL-20AC. YL Clarity software (Prague, Czech Republic), was used to collect and process data. For the chromatographic study, separations were performed on a Kromasil column C-18 (4.6 × 250 mm; 5 μm) (Kromasil, Bohus, Sweden). The mobile phase consisted of ACN (0.2% diethylamine) and water (0.2% diethylamine, adjusted to pH 3 with formic acid), in 16: 84 *v*/*v* ratio. Separation was carried out by isocratic elution chromatography with a detection wavelength set at 310 nm. The flow rate was 1 mL/min, and the temperature of the column oven was set at 35 °C. The injection volume was 5 µL of the respective fraction.

### 3.6. Analysis of Alkaloid and CPC Fractions by GC-MS

Gas chromatography/mass spectrometry (GC-MS) analysis was performed on an Agilent 7890 to GC (Agilent, Palo Alto, CA, USA) with multimodal injector, coupled to an Agilent triple Quad 7000 GC/MS detector. The analysis of the alkaloids was carried out on a capillary column with a stationary phase 5%-phenyl-poly(dimethylsiloxane) HP-5 MS with dimensions 30 m × 0.250 mm I.D × 0.25 µm df, (Agilent J&W, Palo Alto, CA, USA). Helium (99.9999% purity) was used as carrier gas with a flow rate of 0.8 mL/min. The injector temperature was 250 °C, the injection volume of 0.8 µL. The temperature program was initially 100–180 °C (15 °C/min), then increased to 180–300 °C (5 °C/min) and ended with 10 min of heating at 300 °C. The data processing and control of the instrument was performed with the Agilent MassHunter GC/MS software (Version B.05.00/Build 5.0.291.0). Five milligrams of alkaloid crude were dissolved in 500 µL of methanol, codeine standard in methanol was added to obtain a final concentration of 50 µg/mL. Samples were centrifuged for 5 min at 4000 rpm and the supernatant was used for the GC analysis. The alkaloids were identified by comparing their mass spectra and the retention indexes of Kovats (RI), which were calculated taking into account the retention times of C_8_–C_28n_ -paraffin standards, analyzed under the same operational conditions as the samples. In addition, each alkaloid was identified as a function of retention time and the relationship between the intensity of the spectral fragmentation of each of the compounds, for this purpose, three representative ions were taken into account for each alkaloid, one quantitative ion and two qualitative ions. To analyze the fractions obtained in the pH-zone-refinement CPC, they were dried under reduced pressure and reconstituted in 1 mL of methanol for further analysis.

### 3.7. Structural Elucidation of Isolated Alkaloids

The structures of the isolated compounds were elucidated by MS, ^1^H-NMR, ^13^C-NMR, and 2D-NMR. NMR spectra were recorded on a Bruker model Ascend TM, 400 MHz equipped with a liquid probe PABBI 1H/D-BB Z-GRD, inverse, multicore, with automatic tuning with concentration gradient and phase correction. Ten mg of alkaloids were dissolved in CDCl_3_.

### 3.8. Measurement of the Partition Coefficients (KD)

The partition coefficient (KD) was determined according to Ito et al. [37] with slight modifications. Five mg of alkaloid extracts were weighed and dissolved in 3 mL of thoroughly pre- equilibrated upper organic (without TEA as retainer) and lower aqueous (without acid as displacer) phases. The mixture was vigorously shaken in a 10 mL separatory funnel. Once settled, the upper and lower phases were separated and evaporated to dryness. To determine Kacid and Kbasic of each target, the same procedure was performed and acid and TEA were added to the lower and upper phases, respectively. The residues were reconstituted in 1 mL of mobile phase and analyzed by HPLC following the method described above by injecting 5 µL. Based on the ratio of HPLC peak area of each alkaloid in the lower and upper phases the KD values were calculated as follows:

$$KD=\frac{HPLC\;peak\;area\;of\;alkaloid\;in\;upper\;phase}{HPLC\;peak\;area\;of\;alkaloid\;in\;lower\;phase}$$

### 3.9. Purity Determination

The purity was determined by HPLC, dissolving 3 mg of alkaloid in 3 mL of mobile phase and injecting 10 µL of sample, the percentage of purity was calculated with the Equation:

% P = HPLC peak area/HPLC peak total areas × 100

### 3.10. Acetylcholinesterase (AChE) Inhibitory Activity

Acetylcholinesterase inhibitory activity was determined spectrophotometrically using a microplate method described above for Ellman et al. [38] with some modifications. Fifty µL of sample prepared in methanol: 50 mM Tris-HCl buffer pH 8.0 (1:1), were added to a 96-well microplate and serially diluted 10 times. Afterwards, 50 µL of 0.4 U/mL Electrophorus electricus AChE dissolved in 0.1% bovine serum albumin were added and the mixture was stirred and incubated for 30 min at 37 °C. Substrate was prepared as follows: 4 mg of DNTB (5, 5′-dithiobis-(2-nitrobenzoic acid)) and 3, 4 mg of acethylthiocholine iodide (ATCI) were dissolved in 50 mL Tris-HCl buffer with 0.1 M NaCl and 0.02 M MgCl_2_ 6H_2_O. After the incubation time, 100 µL of substrate were added to the mix and the microplate was incubated for an additional 15 min. Readings were performed at 405 nm using galanthamine hydrobromide as a positive control. Graphs and calculation of IC_50_ were performed in GraphPad Prism 6.0.

## 4. Conclusions

Amaryllidaceae species constitute an important source of molecules with enzymatic inhibition and varied biological activity, many of them poorly studied due to the complexity of the crude alkaloid extracts. Purification by pH-zone-refinement CPC proved to be a useful technique for the isolation of alkaloids at large-scale. Hence, in the present work we achieved the isolation of three lycorine alkaloids in a single step from a complex mixture of isoquinoline alkaloids of *R. speciosum* (Amaryllidaceae), all over 84% purity. In addition, we found that one of these alkaloids showed higher AchE inhibition than the clinically used drug, galanthamine.

## Figures and Tables

**Table 1:** GC-MS analysis of the alkaloids from *Rhodolirium speciosum*.

Compound	RI	M^+^	*m*/*z* (Relative Intensity %)
**Lycorine Type**			
11-12-dehydrolycorene	2365	253(62)	253(62), 252(100), 224(11), 166(7,5), 152(6), 139 (7)
Anhydrolycorine	2508	251(52)	250(100), 251(52), 220(1), 201(37), 192(14), 191(17), 165(6), 124(18)
11,12-Didehydroanhydrolycorine	2610	249(64)	249(64), 248(100), 190(25), 163(9), 123(18), 96(36)
Sternbergine	2716	331(23)	331(23), 270(22), 252(12), 229(82), 228(100)
Lycorine	2749	287(26)	268(16), 250(14), 227(78), 226(100), 211(5), 147(12)
Dihydrolycorine	2791	289(36)	288(97), 272(28), 254(40), 214(25), 200(2), 187(22), 162(15), 147(46)
2-*O*-Acetyllycorine	2846	329(18)	328(16), 270(42), 269(62), 268(81), 252(43), 250(100), 227(37), 226(67)
Acetyllycorine derivative	2893	331(43)	330(100), 270(25), 149(7)
Lycorine type alkaloid (2) *	2897	330(<1)	330(1), 312(1), 286(1), 270(1), 253(1), 250(1), 226(<1), 174(<1), 162 (1), 150(1), 125(100), 96(40), 83(1)
Lycorine type alkaloid (3) *	2972	370(<1)	280(1), 254(1), 226(1), 178(1), 147(1), 146(1),150(1), 125(100), 117(1), 108(1), 96(37), 83 (3)
Lycorine type alkaloid (1) *	3045	327(6)	311(1), 285(1), 270(2), 253(3), 226(2), 150(1) 125(100), 108(1), 96(40), 83(7)
**Haemanthamine Type**			
Vittatine	2471	271(82)	272(14), 252(16), 199(89), 187(40), 173(14), 115(22)
8-*O*-Demethylmaritidine	2517	273(100)	230(5), 202(29), 201(74), 189(10), 175(12), 174(31), 129(13), 128(21), 115(46), 56(13)
Aulicine	2616	304(23)	304(23), 288(47), 233(96), 206(60), 190(30), 175(11), 163(23)
Haemanthamine	2634	301(23)	272(100), 257(12), 240(26), 225(16), 211(24), 181(44), 153(13)
**Galanthamine Type**			
Galanthamine	2406	287(77)	287 (41) 286 (47), 244 (10),230 (10), 216 (22), 174 (23), 128 (11), 115 (12)
Norlycoramine	2460	275(57)	274(95), 273(51), 202(53), 188(13), 173(20), 160(29), 115(20)
**Not identified**			
Nerinine type alkaloid (1)	2484	281(1)	271(3), 254(2), 238(1), 207(1), 128(2), 115(2), 109(100), 108(14)
Homolycorine type alkaloid (6)	2401	281(8)	281 (8) 250 (5), 222(4), 147 (23), 129 (100), 112(25), 83 (18), 70 (33), 57(39)
Galantathamine derivate 1	2547	289(97)	289(97), 272(15), 244(10), 230(14), 218(52), 216(17), 174(11), 149(14), 128 (29), 115 (28)
Galanthamine derivate 2	2601	344(100)	345(38), 344(100), 251(2), 248(2), 226(8), 161(7), 147(3), 129(5), 101(6)
Galanthamine derivate 3	2654	306(22)	306(22), 305(100), 290(16), 288(22), 276(27), 248(21), 233(72), 206(47), 175(14)

RI: Values Kovats index. *: isolated alkaloids.

**Table 2:** Different solvent systems used for purification of *Rhodolirium speciosum* alkaloids by pH- zone-refinement centrifugal partition chromatography (CPC).

#	Solvent Systems ^a^	Retentor (TEA) ^b^	Displacer (Acid)
1	*n*-Hept/EtOAc/*n*-PrOH/W	15 mM	HCl 6 mM
2	*n*-Hept/EtOAc/*n*-PrOH/W	15 mM	Acetic acid 6 mM
3	*n*-Hept/EtOAc/*n*-PrOH/W	15 mM	Formic acid 3 mM
4	MtBE/ACN/W	12 mM	Formic acid 6 mM
5	MtBE/ACN/W	15 mM	Formic acid 3 mM

**^a^***n*-Hept: *n*-heptane; EtOAc: ethyl acetate; *n*-PrOH: *n*-propanol; W: water; MtBE: Methyl terbutyl ether; ACN: acetonitrile. ^b^ Triethylamine.

**Table 3:** Partition coefficients of alkaloids 1 and 2 using pH-zone-refinement CPC with the solvent system *n*-Hept/EtOAc/*n*-PrOH/H_2_O (10:30:15:45 *v*/*v*) and different concentrations of acid and TEA.

	Alkaloid 1	Alkaloid 2
K_D_	0.64	0.40
K_basic_ (15 mM TEA)	0.50	0.20
K_acid_ (3 mM formic acid)	24.42	4.38
K_acid_ (6 mM Acetic acid)	30.79	7.39

**Table 4:** Partition coefficients, purity and yields of compounds 1–3 using pH-zone-refinement CPC with the solvent system MtBE/ACN/W, 6 mM formic acid and 16 mM TEA.

	Alkaloid 1	Alkaloid 2	Alkaloid 3
K_D_	2.99	12.24	1.42
K_acid_	33.76	48.65	12.52
K_base_	1.35	0.74	0.43
Dry weight (mg)	65.7	50.1	12.3
Purity (%)	88.2	97.7	84.4
Yield (%)	1.8	9.0	2.2

**Table 5:** ^1^H and ^13^C-NMR data of alkaloids 1, 2, and 3 (*J* in Hz) in CDCl_3_.

	1		2		3	
Position	δ_H_	δ_C_	δ_H_	δ_C_	δ	δ
1	4.66 bs	66.41	5.93 bs	67.50	4.62 s	66.8
2	4.40 bs	80.48	4.15 bs	81.56	4.38 s	80.34
3	5.83 s	119.28	5.58 s	120.05	5.77 s	120.1
4	-	152.68	-	141.98	-	151.7
4a	2.90 d (10)	60.62	2.75 d (10)	66.42	3.90 d (10)	60.7
6α	8.47 s	168.15	4.23 d (13)	56.4	8.55 s	164.7
6β	-		3.70 d (14)	-	-	
6a	-	139.73	-	138.4	-	139.2
7	7.07	109.64	6.96 s	110.08	7.01	109.8
8	-	145.55	-	152.05	-	146.2
9	-	143.48	-	148.20	-	143.1
10	7.51	103.45	7.50 s	108.74	7.49 s	102.9
10a	-	136.93	-	118.5	-	136.5
10b	2.99 bd (10.7)	43.00	2.87 d (10)	42.79	2.99	42.8
11 α,β	2.66 m	27.60	2.77 s	27.46	2.85	27.6
12 α	2.53 dd (15.1, 9.0)	55.77	3.24 m	55.59	3.75	56.1
12 β	3.49 m	-		-		
O-CH_2_-O	6.11 d (1.2)	101.9	6.10 d (1.1)	102.26	6.05 bs	102.2
O-CO-CH_3_	2.30 s	39.66	2.07 s	37.55	2.67	20.9
		161.33		164.38		169.7
O-CO-CH_3_					2.11	21.1
						170.0

## Supplementary Materials

The following are available online at https://www.mdpi.com/2218-1989/10/8/309/s1: Table S1: Different solvent systems used for purification of *R. speciosum* alkaloids by pH zone refinement CPC; Figure S1: pH zone refinement CPC of *R. speciosum* using solvent system: *n*-Hept/EtOAc/*n*-PrOH/H2O (15 mM TEA and 6 mM HCl); Figure S2: pH zone refinement CPC of *R. speciosum* using solvent system: *n*-Hept/EtOAc/*n*-PrOH/H2O (15 mM TEA and 6 mM acetic acid); Figure S3: pH zone refinement CPC of *R. speciosum* using solvent system: *n*- Hept/EtOAc/*n*-PrOH/H2O (15 mM TEA and 3 mM formic acid); Figure S4: pH-zone-refinement CPC of *R. speciosum* using solvent system: *n*- MtBE/ACN/W (12 mM TEA and 6 mM formic acid).
