# Supplementary material for: Isolation of Three Lycorine Type Alkaloids from Rhodolirium speciosum (Herb.) Ravenna Using pH-Zone-Refinement Centrifugal Partition Chromatography and Their Acetylcholinesterase Inhibitory Activities
Source: Metabolites. 2020 Jul 28;10(8):309. doi: 10.3390/metabo10080309 (PMC7465821; doi:10.3390/metabo10080309)
Supplement: Supplementary file 1 [file metabolites-10-00309-s001.pdf]

SUPPLEMENTARY FIGURES

Isolation of three Lycorine type Alkaloids from *Rhodolirium speciosum* using pH-zone-refining Centrifugal Partition Chromatography and their Acetylcholinesterase inhibitory activity

Diana Correa[1], Edgar R. Pastene-Navarrete[1,2,5*], Luis Bustamante[3], Marcelo Baeza[4], Julio E. Alarcón-Enos[2*].

[1]Laboratorio de Farmacognosia, Dpto. de Farmacia, Facultad de Farmacia, P.O. Box 237, Universidad de Concepción, Concepción, Chile.
[2]Laboratorio de Síntesis y Biotransformación de Productos Naturales, Dpto. Ciencias Básicas, Universidad del Bio-Bio, Chillan, Chile.
[3]Dpto. de análisis instrumental, Facultad de Farmacia, Universidad de Concepción, Concepción, Chile.
[4]Dpto. Botánica, Facultad de Ciencias Naturales y Oceanográficas, Universidad de Concepción. Concepción, Chile.
[5]Universidad de Concepción, Unidad de Desarrollo Tecnológico, UDT.

Table 1S. Different solvent systems used for purification of *R. speciosum* alkaloids by pH zone refinement CPC.

#	Solvent Systemns	Retentor (TEA)	Displacer (acid)
1	*n*-Hept/EtOAc/*n*-PrOH/W	15 mM	HCl 6 mM
2	*n*-Hept/EtOAc/*n*-PrOH/W	15 mM	Acetic acid 6 mM
3	*n*-Hept/EtOAc/*n*-PrOH/W	15 mM	Formic acid 3 mM
4	MtBE/ACN/W	15 mM	Formic acid 6 mM
5	MtBE/ACN/W	12 mM	Formic acid 6 mM

n-Hept: n-heptane; EtOAc: ethyl acetate; *n*-PrOH: n-propanol; W: water; MtBE: Methyl terbutyl ether; ACN: acetonitrile.

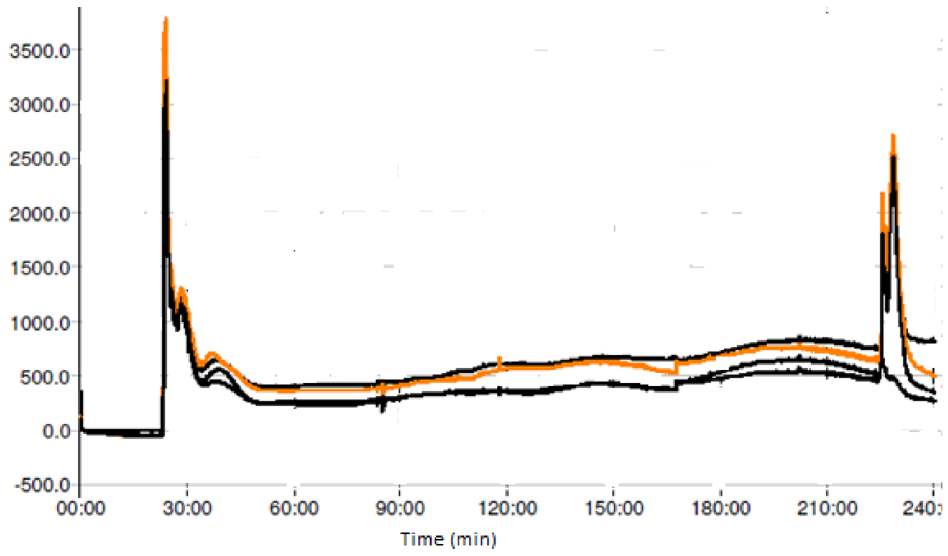

Figure S1: pH zone refinement CPC of *R. speciosum* using solvent system: n-Hept/EtOAc/n-PrOH/H_2O (15 mM TEA and 6 mM HCl).

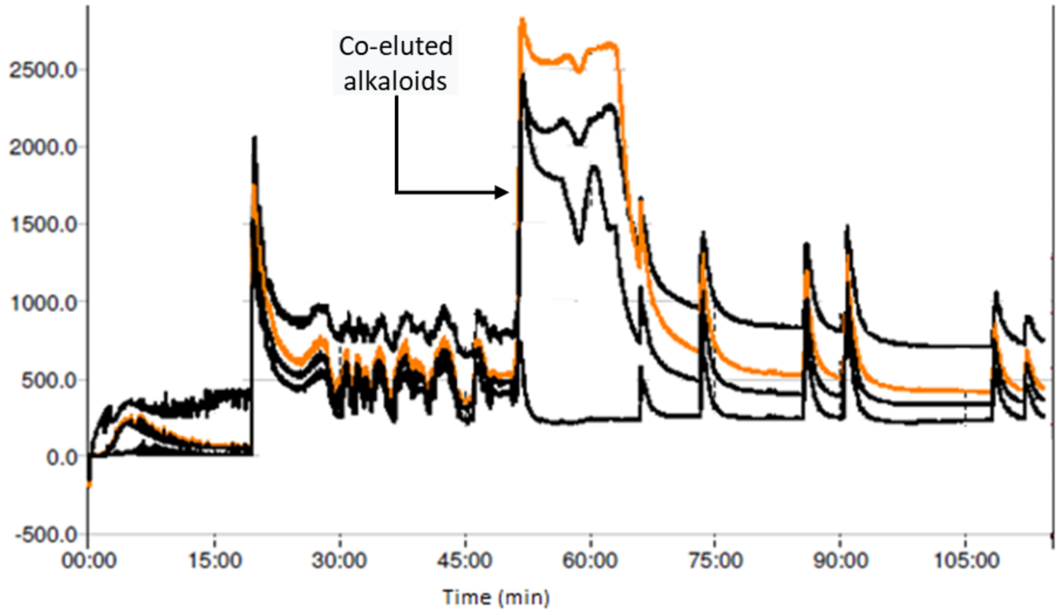

Figure S2: pH zone refinement CPC of *R. speciosum* using solvent system: n-Hept/EtOAc/n-PrOH/H_2O (15 mM TEA and 6 mM acetic acid).

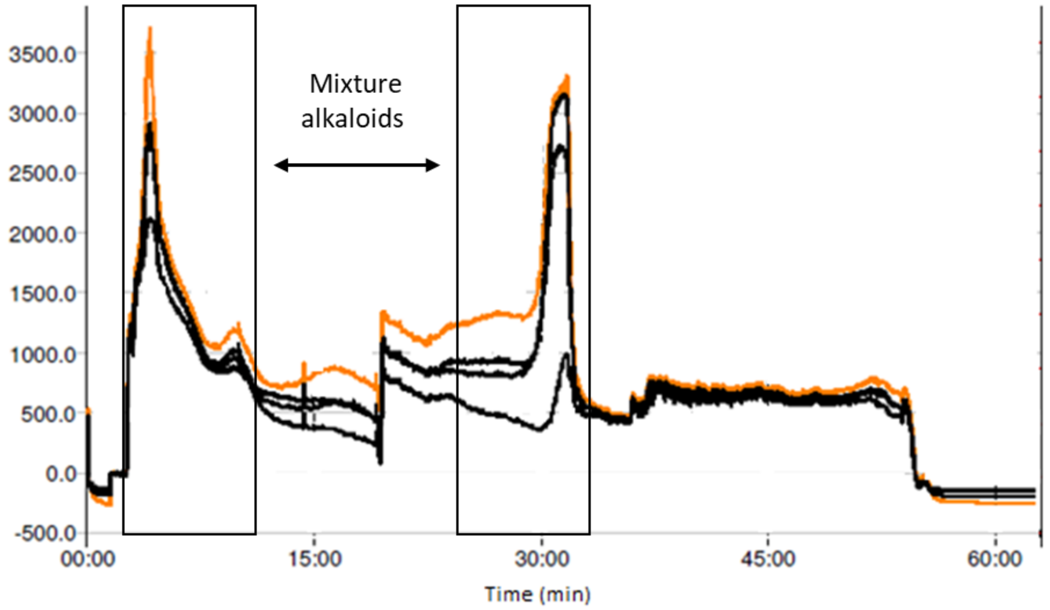

Figure S3: pH zone refinement CPC of *R. speciosum* using solvent system: n-Hept/EtOAc/n-PrOH/H_2O (15 mM TEA and 3 mM formic acid).

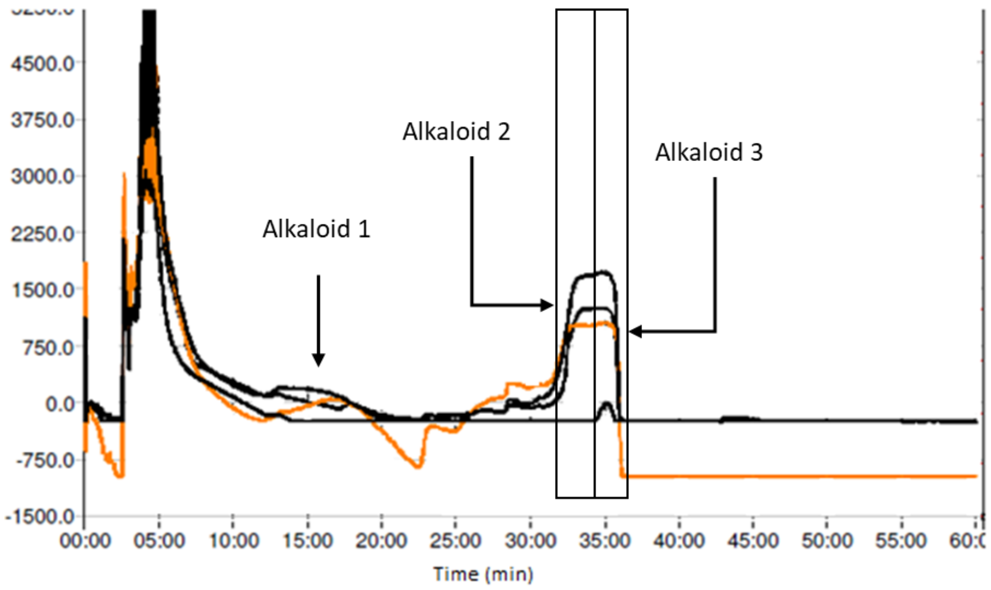

Figure S4: pH zone refinement CPC of *R. speciosum* using solvent system: n- MtBE/ACN/W (12 mM TEA and 6 mM formic acid)
